# Genome-Wide Insights Into the Genes and Pathways Shaping Human Foveal Development: Redefining the Genetic Landscape of Foveal Hypoplasia

**DOI:** 10.1167/iovs.66.12.22

**Published:** 2025-09-09

**Authors:** Callum Hunt, Ha-Jun Yoon, Alvin Lirio, Kayesha Coley, Jun Wang, Nick Shrine, Jianming Shao, Gail D. E. Maconachie, Zhanhan Tu, Jonathan H. Zippin, Pirro G. Hysi, Christopher J. Hammond, Omar A. Mahroo, Mariya Moosajee, Michel Michaelides, Andrew R. Webster, Ala Moshiri, Rui Chen, Martin D. Tobin, Chiara Batini, Mervyn G. Thomas,, on behalf of UK Biobank Eye and Vision Consortium

**Affiliations:** 1 The University of Leicester Ulverscroft Eye Unit, School of Psychology and Vision Sciences, University of Leicester, Leicester, United Kingdom; 2 Department of Population Health Sciences, University of Leicester, Leicester, United Kingdom; 3 University Hospitals of Leicester NHS Trust, Leicester, United Kingdom; 4 Department of Molecular and Human Genetics, Baylor College of Medicine, Houston, Texas, United States; 5 School of Allied Health Professions, Nursing and Midwifery, Faculty of Health, University of Sheffield, Sheffield, United Kingdom; 6 Department of Dermatology, Weill Cornell Medical College of Cornell University, New York, New York, United States; 7 Section of Ophthalmology, King's College London, United Kingdom; 8 Department of Twin Research and Genetic Epidemiology, King's College, London, United Kingdom; 9 Sørlandet Sykehus Arendal, Arendal, Norway; 10 Moorfields Eye Hospital NHS Foundation Trust, London, United Kingdom; 11 UCL Institute of Ophthalmology, University College London, London, United Kingdom; 12 Department of Ophthalmology & Vision Science, University of California Davis School of Medicine, Sacramento, California, United States; 13 Gavin Herbert Eye Institute - Center for Translational Vision Research, Department of Ophthalmology, University of California Irvine, Irvine, California, United States; 14 Department of Genetics and Genome Biology, University of Leicester, Leicester, United Kingdom; 15 Department of Ophthalmology, University Hospitals of Leicester NHS Trust, Leicester, United Kingdom

**Keywords:** fovea, foveal hypoplasia, retinoic acid, CYP26A1, GWAS

## Abstract

**Purpose:**

To define the genetic architecture of foveal morphology and explore its relevance to foveal hypoplasia (FH), a hallmark of developmental macular disorders.

**Methods:**

We applied deep-learning algorithms to quantify foveal pit depth from central optical coherence tomography (OCT) B-scans in 61,269 UK Biobank participants. A genome-wide association study (GWAS) was conducted using REGENIE, adjusting for age, sex, height, and ancestry. Rare coding variants (frequency <1%) were analyzed in an exome-wide rare-variant association study (RVAS). Candidate genes were prioritized using integrative mapping; pathway, cross-ancestry, and genetic-correlation analyses were exploratory.

**Results:**

GWAS identified 126 sentinel variants, including 47 novel associations. Integrative mapping prioritized 129 putative causal genes, with 64 not previously implicated in foveal biology. Enriched pathways included retinoic acid metabolism (e.g., *CYP26A1*), photoreceptor differentiation (e.g., *VSX2*), extracellular matrix organization, and pigmentation. RVAS identified missense variants in *ACTN3* and *ESYT3* (*P* < 5 × 10^−^⁹) associated with FH features. Polygenic scores were predictive across African and South Asian ancestries. Overlap was observed with monogenic FH genes (*TYR*, *OCA2*, *PAX6*, *AHR*) and with genes underlying systemic diseases (*COL11A1, KIF11, TUBB4B, PHYH*). Re-examination of OCTs in affected individuals confirmed FH in select cases, including those with recurrent *TUBB4B* p.(Arg390Trp) variants.

**Conclusions:**

This is the first GWAS of human foveal morphology. Our findings redefine the genetic and biological framework underlying normal foveal development and foveal hypoplasia (FH). By linking common variation to rare monogenic disease, we establish a continuum model of FH with implications for future mechanistic and clinical investigation.

High-acuity central vision relies on the specialized anatomy of the fovea, a pit-like depression at the macular center in which inner retinal layers are displaced and cone photoreceptors are densely packed.^1^ Foveal morphogenesis begins in utero and continues for several years post-natally, culminating in a rod-free, cone-rich zone with a well-defined pit.^1^^–^^4^ Arrest of this process results in foveal hypoplasia (FH), characterized on optical coherence tomography (OCT) by a shallow or absent pit and persistence of inner layers.^5^^,^^6^ Clinically, FH is common in albinism and is often accompanied by infantile nystagmus, chiasmal misrouting, and reduced visual acuity.^7^^–^^9^

To date, the best-established genetic causes of FH involve pigment pathways^10^ (e.g., *TYR*, *OCA2*, and other albinism-related genes), yet ∼30% of patients with albinism-like FH remain genetically unsolved.^11^^–^^13^ Moreover, FH is reported in cone dysfunction syndromes (e.g., achromatopsia)^8^^,^^14^^,^^15^ and in a broad range of inherited retinal diseases (e.g., *CRB1*), where its presence may correlate with poorer vision.^16^ These observations suggest that additional developmental mechanisms, beyond pigmentation, shape foveal architecture.

OCT enables in-vivo visualization of the macula^17^ and is the reference standard for diagnosing and grading FH.^5^^,^^8^ Quantitative OCT traits, especially retinal-layer thickness, have proven powerful endophenotypes in genome-wide association studies (GWAS), yielding numerous loci for macular structure and disease.^18^^–^^23^ However, the genetic basis of the foveal pit itself, a defining feature that discriminates normal from hypoplastic foveae, has not been interrogated on a genome-wide scale.

Here, we address this gap by performing the first large-scale GWAS of foveal pit depth. Using deep-learning algorithms, we quantified pit depth on central OCT B-scans from 61,269 UK Biobank participants and tested common variants for association. We then integrated 12 complementary variant-to-gene mapping strategies, executed an exome-wide rare-variant association study, and explored pathway enrichment, cross-ancestry association testing and genetic correlations with ocular traits. By framing the results against known monogenic causes of FH and systemic conditions with foveal involvement, we aim to (i) redefine the genetic landscape of FH and foveal development, (ii) identify biological pathways beyond pigmentation and, (iii) provide a foundation for future diagnostic and therapeutic approaches to disorders featuring foveal maldevelopment.

## Methods

### UK Biobank Data and OCT

Phenotypic and genetic data available from UK Biobank (UKB) were accessed and analyzed as part of this research, under UKB application 85881. UKB has approval from the North West Multi-Centre Research Ethics Committee (MREC) as a Research Tissue Bank approval (REC reference: 21/NW/0157).

The UKB is a large longitudinal cohort study which recruited approximately 500,000 people aged between 40–69 years across the UK.^24^ In addition to collecting baseline participant characteristics, a subset of participants underwent ophthalmic assessments including OCT examination. OCT scans in UK Biobank were obtained using the TOPCON 3D OCT1000 Mark 2 instrument, which constructs a three-dimensional scan of the retina in a 6 × 6 mm^2^ area, by combining 128 cross-sectional B-scan images per eye across the macula.^25^ Foveal pit depth was quantified using a deep-learning algorithm: a ResNet-50 model was trained to detect key landmarks on the foveal B-scan (the pit floor and the rim peaks) (Supplementary Fig. S1). Pit depth was defined as the vertical distance from the central pit to the average height of the two rim peak points. Strict quality control excluded scans with poor quality or low landmark prediction confidence (<0.99) to ensure reliable phenotypes.

### Genotyping and Genome Wide Association Study

Participants were genotyped using array-based platforms and imputed using a high-coverage reference panel (Genomics England). Genetic ancestry was determined by principal components and *k*-means clustering^26^ with the 1000 Genomes reference,^27^ and only European-ancestry individuals were included in the primary GWAS to minimize population stratification. GWAS was then performed using REGENIE^28^ (v3.4.1), a two-step GWAS software that uses whole genome regression to control for population structure. In step 1 of regenie, we used genotyping array variants meeting the recommended quality control criteria (<10% genotype missingness, minor allele frequency [MAF] > 1% and Hardy-Weinberg equilibrium *P* > 1 × 10^−^^15^). For step 2, we performed association testing on imputed genetic variants with minor allele count > 10 and used an INFO score (imputation quality) filtering procedure (INFO ≥ 0.3 for MAF ≥ 0.01, and INFO ≥ 0.8 for MAF < 0.01). Association testing included covariates age, sex, height, and the top 20 genetic principal components. Genome-wide significance was *P* < 5 × 10^−^^8^.

### Rare Variant Association Study

To identify additional genetic contributors beyond common variants, we conducted an exome-wide rare variant association study (RVAS) using REGENIE (v3.4.1) and the same co-variates as the GWAS. Rare-variant analyses were performed using UK Biobank whole exome-sequencing (WES) data in participants with OCT imaging. After QC, 59,313 individuals contributed to RVAS (versus 61,269 in the GWAS). UKB WES protocols and analysis pipelines have previously been described (UKB showcase category ID: 170, available at https://biobank.ndph.ox.ac.uk/ukb/label.cgi?id=170).^29^ Both single rare variants (MAF < 1%) and gene-based collapsing tests for protein-altering variants were assessed using REGENIE^28^ (v3.4.1). We used the RVAS findings in two ways: (i) to assist in the identification of causal genes near GWAS signals, and (ii) for the discovery of genes not nearby GWAS signals. For (i) we used a suggestive threshold of *P* < 5 × 10^−6^ for single-variant tests and *P *< 2.9 × 10^−6^ for gene-based tests (Bonferroni-adjusted for ∼18,000 genes). For (ii) we included a more stringent discovery threshold (*P* < 5 × 10^−^⁹), and reviewed OCT images of variant carriers to explore potential anatomical correlates.

### Fine Mapping and Variant-to-Gene Mapping

Genome-wide significant loci (*P* < 5 × 10^−8^) were fine-mapped using PolyFun-SuSiE^30^ or the Wakefield method,^31^ which identified the most likely causal variants (sentinel variants) ±1 Mb of lead signals. We next determined whether these sentinel variants were previously reported or in linkage disequilibrium (LD, *r*^2^ > 0.1) with signals from retinal layer GWAS.

Sentinel variants were then mapped to candidate genes using 12 complementary criteria (Supplementary Fig. S2). Evidence is grouped into positional (proximity to genes and gene-features), in-silico pathogenicity prediction, RVAS (single-variant and gene-based tests), disease-informed (Orphanet or MGI database search), and regulatory. For regulatory support we considered quantitative trait loci (QTLs), polygenic priority scores, and *cis*-regulatory element mapping. For QTLs we considered bulk human retina eQTLs,^32^ and blood plasma pQTLs from two sources: UK Biobank (Olink; 2923 proteins)^33^ and deCODE Genetics (4719 proteins via 4907 aptamers).^34^ Significance thresholds were *P* < 5 × 10^−8^ (UKB) and *P* < 1.8 × 10^−9^ (deCODE). Polygenic priority scores were calculated by integrating GWAS summary statistics with 57,543 gene features, including gene expression, pathways, and protein–protein interactions.^35^ For *cis*-regulatory element mapping we used a multiomics single-cell dataset from developing human retina^36^ that combined chromatin accessibility and gene expression to link *cis*-regulatory elements to target genes.

### Gene Follow-Up and Pathway Analysis

After synthesizing the variant-to-gene evidence, we defined two gene-sets for downstream analysis: (i) a set of putative causal genes (*n* = 129), comprising those supported by at least two independent lines of evidence, and (ii) a broader set of implicated genes (*n* = 364), encompassing all genes supported by at least one criterion.

Putative causal genes (gene-set i) were first manually investigated using online databases (Supplementary Table S5) and assigned to functional groups related to pigmentation/RPE, metabolism, photoreceptors, retinal cell fate, retinal vasculature, or cytoskeleton/ECM. To validate and extend these findings, we performed pathway enrichment analysis using Metascape,^37^ which integrates GO-term enrichment with multiple curated pathway databases. Pathways were prioritized based on significance at a false discovery rate < 5%.

For the expanded list of genes (gene-set ii), we re-examined macular OCT scans from individuals with molecularly confirmed systemic diseases within the Moorfields Eye Hospital (MEH) dataset, prioritizing those with rare pathogenic variants in genes from the expanded list showing potential relevance to FH. We identified patients with biallelic *PHYH* variants (*n* = 13), and heterozygous variants in *KIF11* (*n* = 9), *COL11A1* (*n* = 6), and *TUBB4B* (*n* = 8) and reviewed their OCT scans. Patients identified at MEH were independent of UK Biobank participants (i.e., no overlap). MEH cases carried rare, pathogenic variants in genes implicated by our GWAS/RVAS and were ascertained clinically; this contrasts with the common variants and population signals discovered in UKB.

### Cross-Trait Analyses

To assess shared genetic architecture, we used LD score regression^38^ to quantify genetic correlations between foveal pit depth and other traits, including age-related macular degeneration, glaucoma, refractive error, and pigmentation-related phenotypes (Supplementary Table S6).

### Cross-Ancestry Association Analysis

To assess the transferability of our primary GWAS findings in the European (EUR) cohort across ancestries, we calculated polygenic scores in both African (AFR) (*n* = 1819) and South Asian (SAS) (*n* = 2134) participants. These scores were generated using PRS-CS,^39^ trained on European summary statistics, and evaluated in non-European groups via linear regression adjusted for the same co-variates used in the GWAS.

## Results

### GWAS of Foveal Pit Depth

**Figure 1.**
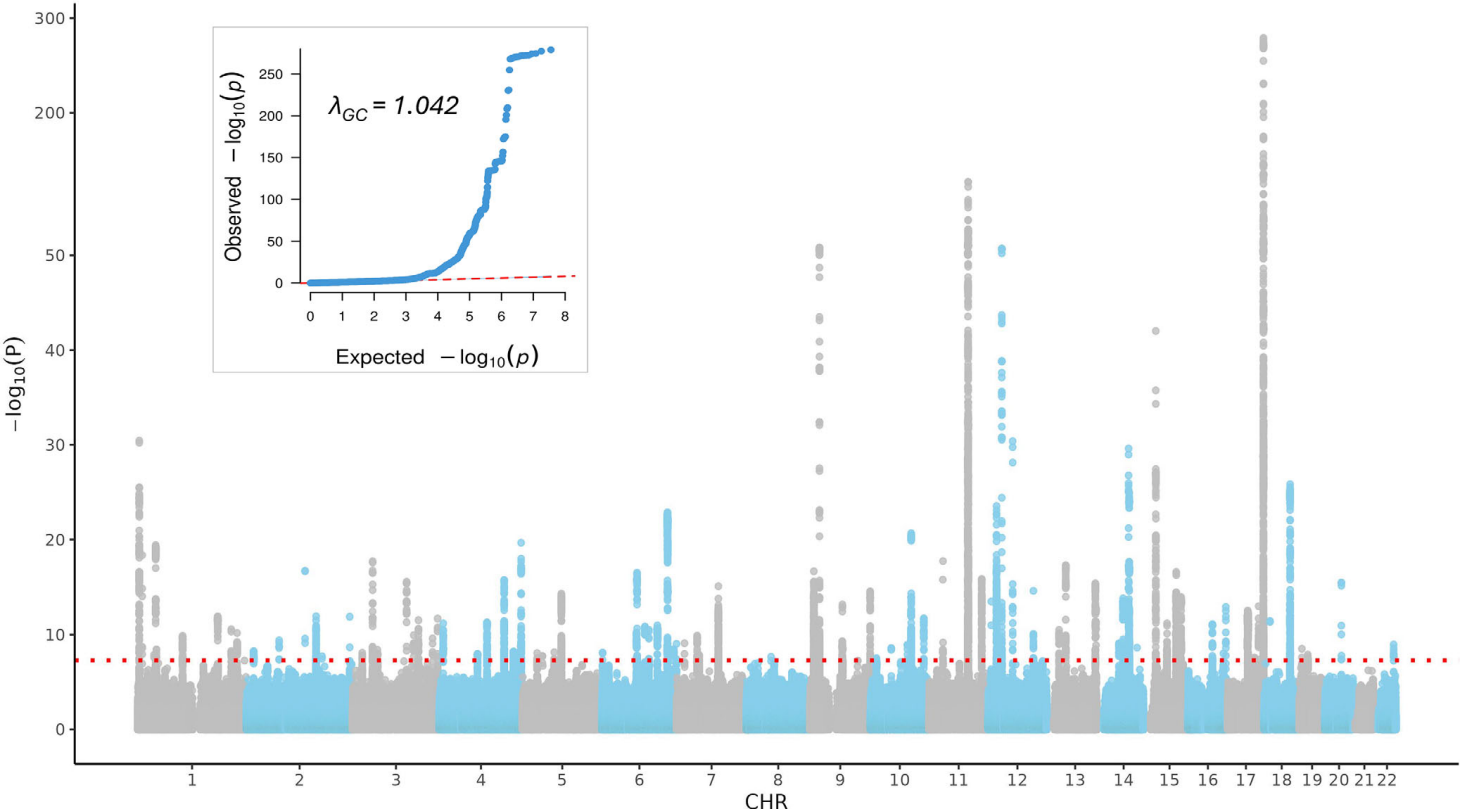
Manhattan and quantile-quantile (QQ) plots for genome-wide association analysis of foveal pit depth. The Manhattan plot displays genome-wide association results, with each point representing a genetic variant. The x-axis indicates chromosomal position, and the y-axis shows the −log_10_ (*P* value). The *red dotted lines* represent the genome-wide significance threshold (*P* < 5 × 10^−8^). The QQ plot compares observed versus expected −log_10_ (*P* values) under the null hypothesis, with the genomic inflation factor (λ_GC_) provided as a measure of population stratification or confounding.

In 61,269 individuals of European ancestry, the median pit depth was 116.4 µm (range 2.7–231.1 µm). GWAS of 35.8 million variants (Fig. 1) identified 126 sentinel variants implicated by lead signals (Supplementary Tables S1, S2), 47 of which were not previously associated with macular structure (Supplementary Table S3). SNP-based heritability of foveal pit depth was ∼0.29 (SE = 0.03), indicating that nearly 30% of trait variance is explained by common variants.

### Gene Prioritization and Biological Pathways

**Figure 2.**
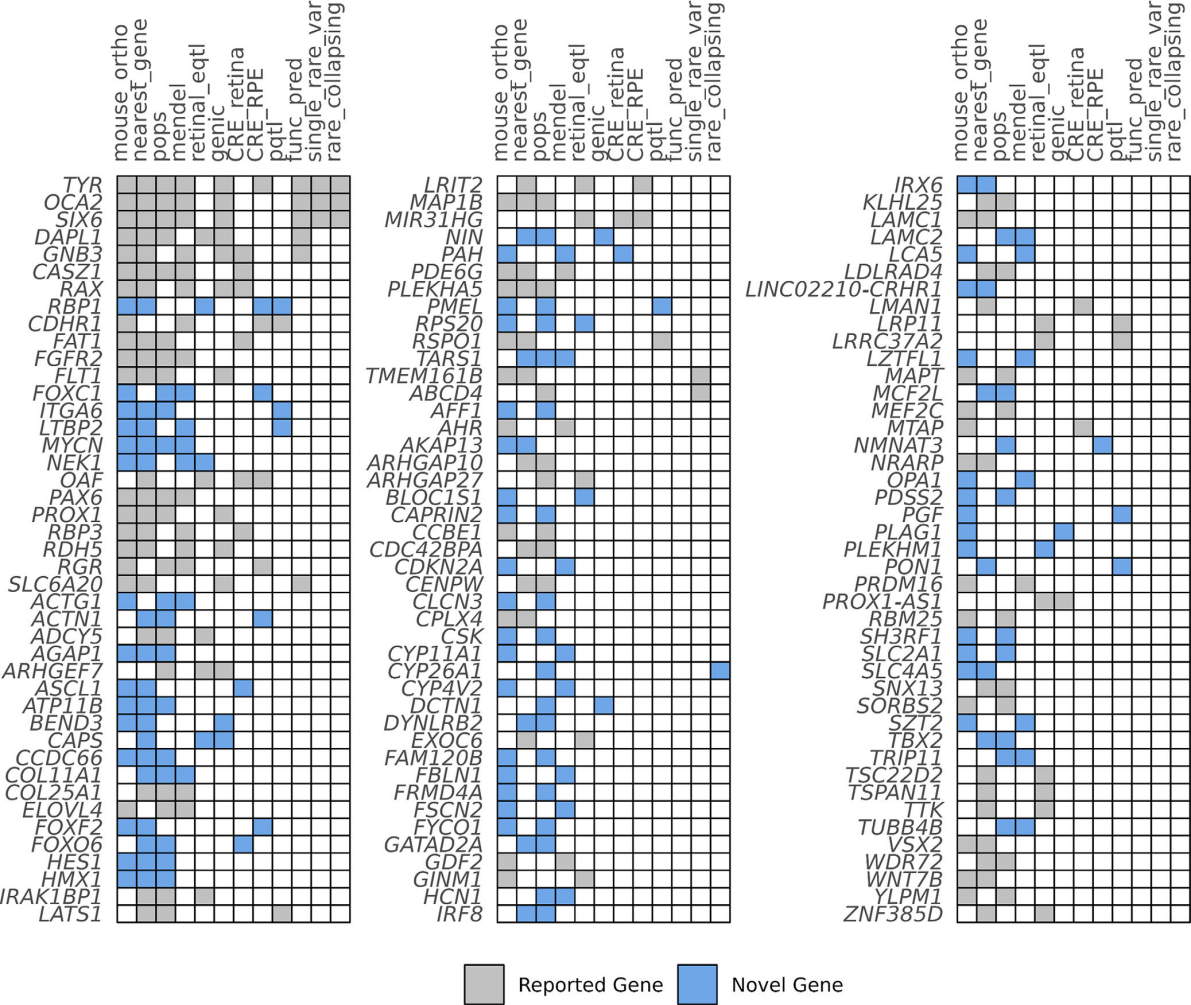
Summary of the variant-to-gene evidence supporting putative causal genes. Evidence for novel gene associations with foveal pit depth is highlighted in *blue*, whereas genes previously reported in GWAS of the macular region or associated with known foveal diseases are shaded in *gray*. Columns are ordered by the number of genes implicated by each line of evidence.

**Figure 3.**
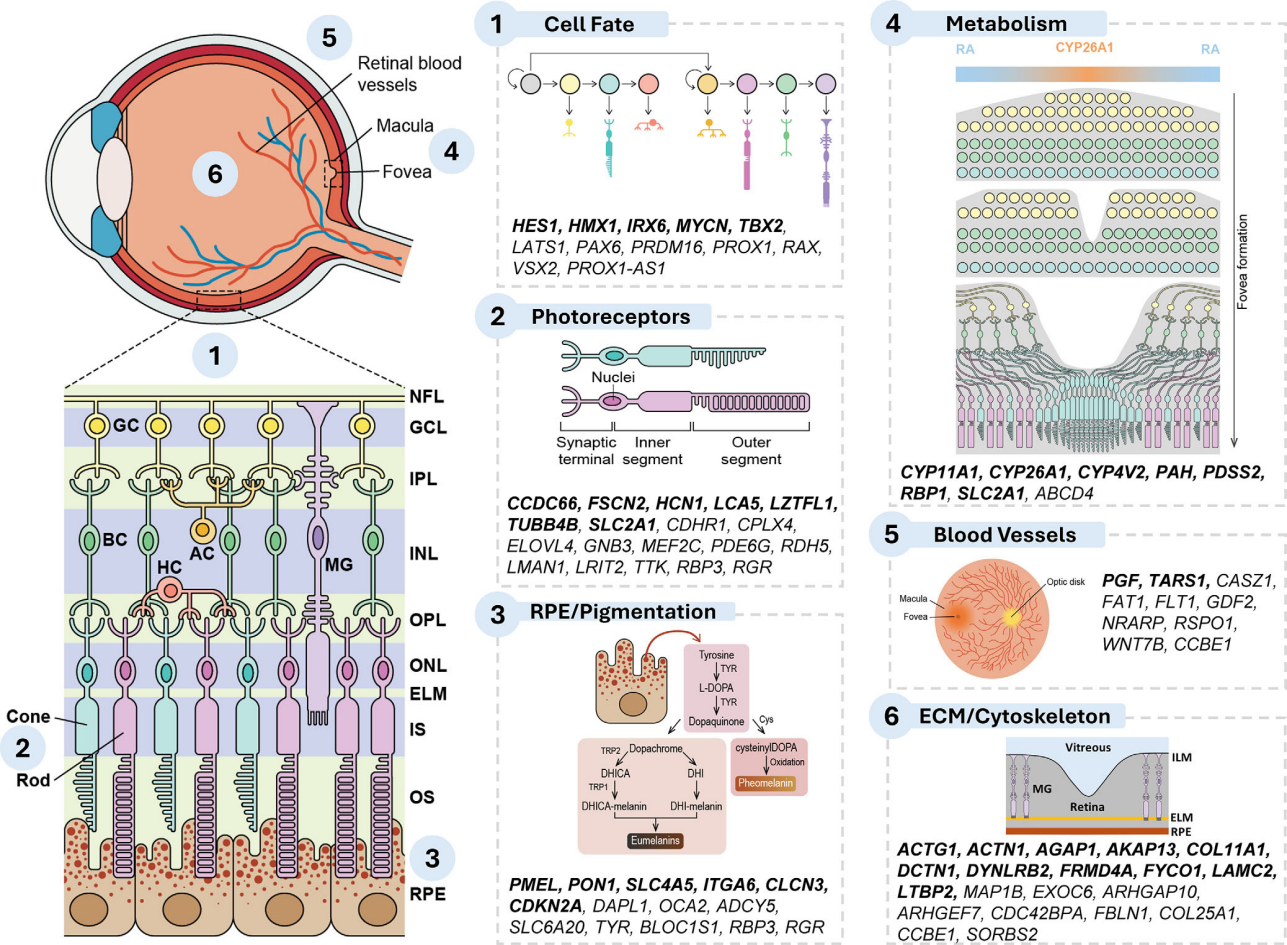
Schematic representation of the retina and foveal region within the human eye. Putative causal genes relevant to prioritized functional groups involved in foveal development are displayed alongside their respective functional categories. Genes highlighted in bold are novel gene associations not reported in previous GWAS of the macular region or as foveal disease genes.

Using 12 complementary lines of evidence, we identified 364 genes linked to foveal development across all loci (Supplementary Table S4). From these, we prioritized 129 genes that are supported by at least two lines of evidence (putative causal genes), including 64 novel to foveal development (Fig. 2). We then analyzed the biological pathways related to the putative causal genes (Supplementary Table S5, Fig. 3). Pathways implicated included retinoic acid metabolism (e.g., *CYP26A1*), photoreceptor and retinal cell fate determination (e.g., *VSX2, RBP3*), cytoskeletal/extracellular matrix organization (e.g., *LAMC1*), and pigment biology (e.g., *OCA2, PMEL*). Overlap was observed with Mendelian FH genes (e.g., *TYR*, *OCA2*, *PAX6* and *AHR*). Genetic correlation with refractive error was present (*r*_g_ = 0.16, *P* = 3.17 × 10^−10^) but not macular degeneration or glaucoma.

### RVAS

**Figure 4.**
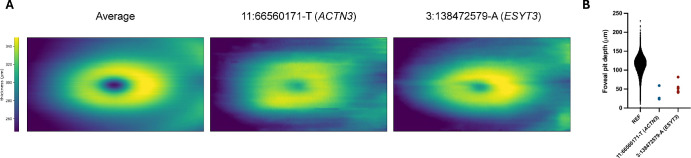
Abnormal foveal morphology in individuals carrying rare genetic variants. (**A**) Heatmaps showing average retinal thickness across the central macula, measured from the internal limiting membrane (ILM) to Bruch's membrane. The *left panel* shows the mean retinal thickness map for the broader study cohort of European ancestry. The *middle* and *right panels* show retinal thickness maps from individuals heterozygous for rare variants in ACTN3 (11:66560171-T) and ESYT3 (3:138472579-A), respectively. Compared to the average map, both variants are associated with a shallower and more flattened foveal pit contour. (**B**) Violin plots comparing foveal pit depth in the general cohort (*black*) versus individuals carrying the ACTN3 (*blue*) and ESYT3 (*red*) rare variants. Both variants are associated with a significant reduction in pit depth, consistent with abnormal foveal development.

Two rare (MAF <1%) missense variants met our predefined rare-variant discovery threshold (*P* < 5 × 10^−9^): *ACTN3* (11:66560171-T) and *ESYT3* (3:138472579-A). Carriers showed significantly shallower pits (variant effect size up to −69 µm) (Fig. 4) with inner retinal layer continuation on OCT, consistent with FH.

### Cross-Ancestry Analysis

Foveal pit depth was significantly deeper in African (median: 124.4 µm) and South Asian (119.5 µm) participants compared to Europeans (AFR vs. EUR [*P* = 8.07 × 10^−61^]; SAS vs. EUR [*P* = 1.36 × 10^−11^]). Polygenic scores trained on European data were significantly associated with pit depth in both populations (South Asian β = 4.90, *P* = 3.9 × 10^−30^; African β = 2.62, *P* = 1.6 × 10^−8^), supporting shared genetic architecture.

### Phenotypic Lookup of GWAS Genes Implicated in Systemic Disease

**Figure 5.**
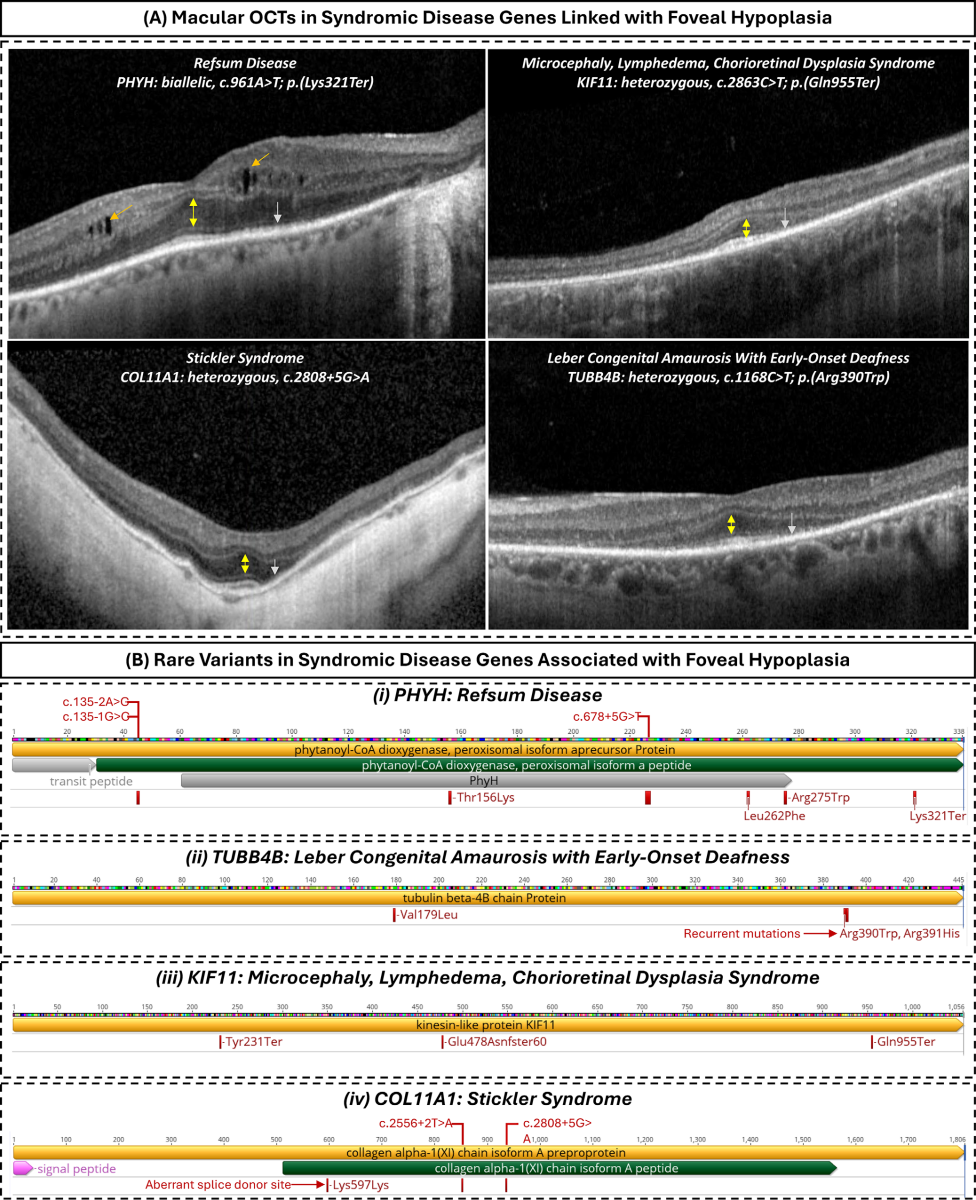
(**A**) Macular OCT scans from individuals with molecularly confirmed systemic or retinal syndromes demonstrate foveal architectural disruption and outer retinal abnormalities. In Refsum disease due to a *PHYH* nonsense mutation, the scan shows a shallow foveal pit, cystic changes in the inner retina (*orange arrows*), and loss of the inner segment ellipsoid (ISe) band (*green arrow*). In *KIF11*- and *COL11A1*-associated syndromes, the foveal pit is absent, with accompanying ISe loss (*gray arrows*). In the *TUBB4B*-associated case, a shallow indentation is present at the fovea. Across all examples, widening of the outer nuclear layer at the foveal center (*yellow arrows*) helps localize the fovea, despite the absent or shallow pit. The intrusion of inner retinal layers at the foveola, combined with a shallow or absent pit, is a hallmark of FH, although outer retinal abnormalities are also present in all cases. (**B**) Schematic protein models showing locations of rare variants observed in our cohort for PHYH, TUBB4B, KIF11, and COL11A1. Variants are mapped along the canonical protein sequence with relevant annotations. Predicted splice-site variants are shown above each protein track, positioned according to their anticipated impact on coding sequence, while missense, nonsense, and frameshift variants are shown below. *TUBB4B* shows a notable recurrent missense variant p.(Arg390Trp) in multiple individuals with FH. The *COL11A1* synonymous variant (Lys597Lys) activates an aberrant splice donor site. The mapping illustrates diversity of variants and location of recurrent variants.

To explore broader clinical relevance, we examined the extended list of 364 genes supported by at least one variant-to-gene criterion. Several of these genes are implicated in systemic disorders that can feature retinal involvement, including *KIF11* (microcephaly–lymphedema–chorioretinal dysplasia), *COL11A1* (Stickler syndrome), *PHYH* (Refsum disease), and *TUBB4B* (Leber congenital amaurosis with early-onset deafness). Re-examination of OCT scans from individuals with these diagnoses in a tertiary ocular genetics clinic revealed shallow foveal pits, FH or disrupted architecture (Fig. 5, Supplementary Table S7). None of the cases had been coded as FH before our targeted review, likely because outer retinal/photoreceptor abnormalities dominate the clinical picture in these syndromes; FH may be overlooked without focused OCT reassessment. Recurrent *TUBB4B* mutations (c.1168C>T, p.(Arg390Trp)) was consistently associated with FH, highlighting a potential genotype–phenotype correlation. Taken together with the GWAS results, this suggests that common variation at these loci may influence foveal structure or modulate phenotypic expressivity in developmental macular disorders.

## Discussion

Our study provides the first comprehensive genetic dissection of human foveal pit architecture, revealing new biological mechanisms that shape foveal development and extending the clinical understanding of FH. By combining deep-learning based phenotyping of foveal pit depth with large scale genome-wide and rare variant association analyses, we identified 64 novel genes not previously implicated in foveal or macular structure. Rare-variant analysis uncovered two genes (*ACTN3* and *ESYT3*) in which rare protein-altering variants were associated with significantly shallower foveal pits and OCT features of FH, underscoring the contribution of both common polygenic and rare large-effect variation to foveal morphology. These findings expand the genetic landscape of FH beyond classical pigment-related pathways (e.g., *TYR*, *OCA2*), implicating a diverse array of developmental processes including retinoic acid metabolism, vascular patterning, cytoskeletal organization, and retinal cell fate specification. Several of the genes identified in our study are also implicated in systemic diseases, some of which demonstrated FH on OCT re-evaluation. Although FH has previously been reported in *KIF11*-related microcephaly syndromes^40^ and Stickler syndrome (*COL2A1*),^41^ our data provide the first clinical evidence linking *PHYH* (Refsum disease), *COL11A1*-related Stickler Syndrome and *TUBB4B* (Leber congenital amaurosis with early-onset deafness) with FH.

Our findings highlight the role of retinoic acid (vitamin A derivative) signaling in foveal development. This pathway had not been implicated in human foveal formation prior to our study. We found that genes involved in retinoic acid metabolism and gradients (e.g., *CYP26A1*, a retinoic acid-degrading enzyme) are associated with pit depth. Interestingly, *CYP26A1* was implicated by both our GWAS and RVAS. This aligns with experimental evidence from model systems: in chicks, establishing a retinoic acid-free zone is required to pattern the high-acuity foveal region,^42^ and in retinal organoids, low retinoic acid levels promote cone-rich (as opposed to rod) development.^43^ In mice, Cyp26a1 (and Cyp26c1) help create retinoic acid gradients across the retina.^44^ Our human genetic results suggest that similar retinoic acid–mediated patterning occurs in the developing fovea, potentially influencing the high cone density and absence of rods at the foveal center. The implication is that modulating retinoic acid signaling (for instance, via *CYP26A1* or related proteins) could influence foveal architecture and normal foveal formation.

We also discovered multiple developmental transcription factors and cell-fate regulators as important for foveal structure. Notably, we implicate two novel developmental regulators, *HES1* and *HMX1*, which are expressed in retinal progenitor cells and direct the spatio-temporal patterning.^45^^,^^46^ *HES1*, a Notch effector, is known to maintain retinal progenitors in an undifferentiated state, modulating the timing of neurogenesis. *HMX1* (also known as NKX5-3), a homeobox transcription factor expressed in both the developing retina and ear, governs regional retinal patterning. Biallelic variants have been linked to oculo-auricular syndrome, with associated phenotypes including congenital nystagmus, FH, and coloboma.^46^ The expression of *HMX1* in the human fetal retina is polarized toward the temporal and posterior regions, aligning with the anatomical location of the fovea.^46^ These findings support a role for *HMX1* in the spatial organization and maturation of the central retina, and our data now implicate common variation at this locus in determining foveal architecture. Taken together, our findings suggest that the precise ratio and arrangement of diverse retinal cell types, not only photoreceptors, may be essential for sculpting the foveal morphology, reinforcing a model in which cell fate specification intersects with mechanical and metabolic factors to determine foveal architecture.

Consistent with clinical knowledge, we found several common variants in melanin-related genes (e.g., *TYR*, *OCA2*) associating with pit depth. Individuals with albinism, caused by pathogenic variants in these genes, often have FH,^5^^,^^8^ but our results show that even population-level variation in these loci can modulate foveal structure. Supporting this, recent work has shown that certain hypomorphic *TYR* alleles common in European populations contribute to “missing” heritability in mild or undiagnosed albinism.^12^^,^^13^ Moreover, a recent prospective deep-phenotyping study demonstrated that up to 32% of individuals heterozygous for known albinism-causing variants (carriers) exhibit detectable FH despite lacking a clinical diagnosis.^47^ We also identified novel pigment-related genes such as *PMEL* (involved in melanosome biogenesis) and *BLOC1S1* (a component of the biogenesis complex for lysosome-related organelles, including melanosomes), further supporting the role of melanosome formation and transport, key features of syndromic albinism, in foveal development. These findings reinforce that proper RPE melanosome function is critical for foveal pit formation, and that even mild perturbations in pigment biology can influence foveal anatomy. This raises the intriguing prospect that therapies aimed at enhancing RPE melanin content or melanosome function might offer developmental or functional benefits if applied early in life.

Another key finding is the involvement of extracellular matrix (ECM), cytoskeletal, and vascular development pathways in determining foveal morphology. We highlight genes such as *ACTN3*, typically associated with muscle fiber composition, now implicated in foveal morphology via a rare variant of large effect. This, along with other ECM- and cytoskeleton-related loci (e.g., collagen and laminin family members), suggests that the biomechanical properties of the retina, including its elasticity, cellular adhesion, and structural support, play an important role in shaping the fovea. Several of the implicated genes also point to a role for vascular patterning. The foveal avascular zone and centrifugal displacement of inner retinal layers are known to be crucial for pit formation,^48^ and clinical conditions such as retinopathy of prematurity^1^ and familial exudative vitreoretinopathy,^40^ including *KIF11*-related syndromes, provide strong evidence that impaired retinal vascular development leads to FH. Our genetic findings underline this concept by implicating molecules involved in both vasculogenesis and ECM remodeling, potentially mediating mechanical forces that shape the fovea during development. These results suggest that foveal architecture is not only influenced by pigmentary and neurodevelopmental cues, but also by vascular regression and biomechanical tension within the retina.

We observed overlap between the genes identified in this GWAS and genes known to cause Mendelian disorders featuring FH. For instance, signals in or near albinism related genes (*TYR*, *OCA2*), *PAX6* (aniridia and other eye development disorders), *AHR* (recently identified to cause recessive FH with nystagmus),^49^ *KIF11* (microcephaly with or without chorioretinopathy, lymphedema, or impaired intellectual development), *COL11A1* (Stickler syndrome), and others suggest that the spectrum from normal to diseased fovea is partly determined by load of common variants in these pathways. In other words, the same biological processes can be disrupted by a single high-impact variant (causing a syndrome) or by the cumulative effect of many mild variants (altering the foveal architecture). This supports a continuum model of foveal development and disease. A practical implication is the concept of polygenic modifiers: patients with monogenic FH conditions might have varying foveal phenotypes and visual outcomes in part because of their polygenic background. This paradigm is well-established in other genetic disorders where common variants modify disease expressivity.^50^ Additionally, our findings may help explain aspects of missing heritability in FH-associated disorders,^13^ particularly in cases where a clear monogenic cause is not identified, potentially moving towards an oligogenic pattern of inheritance. Our results suggest that combinations of rare and common variants, acting additively or epistatically, could account for undiagnosed or milder phenotypes in suspected genetic FH cases.

Despite overlap with disease genes, we observed little genome-wide genetic correlation between pit morphology and late-onset complex eye diseases such as AMD or glaucoma. This implies that, aside from specific loci, the overall polygenic background influencing foveal morphology is distinct. The main exception was a modest correlation with refractive error: alleles associated with shallower foveae also conferred risk of hyperopia. Clinically, this aligns with the observation that smaller eyes, as in hyperopia, nanophthalmos, or microphthalmia, often exhibit underdeveloped foveae, suggesting shared developmental mechanisms influence both axial growth and foveal formation.

We acknowledge limitations of our study. First, our GWAS was conducted in a single cohort and lacks an independent replication sample. While the associations we report are highly significant and biologically plausible (and many are supported by prior knowledge of eye development), replication in other populations would strengthen confidence in each specific locus. Unfortunately, few datasets worldwide currently have both high-quality OCT imaging and genomic data in large numbers. Second, the UK Biobank cohort is predominantly of European ancestry; thus our findings have maximum relevance to European ancestry groups. We did attempt to validate association results in African and South Asian subsets using PGS analysis, with encouraging evidence of shared effects. However, both groups had small sample sizes, and further analysis is needed to identify population-specific genetic factors and ensure insights benefit all ancestries. Future studies in more diverse cohorts are needed to uncover population-specific genetic factors and to ensure that genetic insights benefit all ancestries. Third, our phenotype is foveal pit depth from the central OCT B-scan, an informative quantitative endophenotype, but a clinical diagnosis of FH requires persistence of inner retinal layers at the foveola and cannot be inferred from pit depth alone; accordingly, we did not assign FH case labels or exclude participants on that basis. Another limitation is that our variant-to-gene mapping, while comprehensive, may have erroneously prioritized some genes and missed others. Because the data point to many plausible targets per locus, functional experiments will be required to pinpoint the causal genes and mechanisms. Finally, our focus was on discovery of genetic loci; we did not directly examine how environmental factors or gene–environment interactions might also play a role in foveal development. These aspects remain open for investigation.

In conclusion, this study substantially advances our understanding of the genetic architecture of the human fovea by identifying over 120 sentinel variants and prioritizing many novel genes associated with foveal pit depth, a core feature of foveal maturation. These findings reveal that foveal development is governed by a complex interplay of biological pathways including retinoic acid signaling, neuronal patterning, extracellular matrix dynamics, cytoskeletal organization, and pigment metabolism within the retinal pigment epithelium. Importantly, many of these pathways are also disrupted in Mendelian syndromes characterized by FH (e.g., *TYR, OCA2, PAX6, KIF11, AHR*), supporting a continuum model of foveal development in which the same developmental programs may be subtly perturbed by common variants or severely disrupted by rare, high-impact mutations. Beyond known eye-specific genes, our findings also implicate novel systemic disease genes (e.g., *PHYH*, *COL11A1, TUBB4B*), expanding the landscape of disorders associated with FH and reinforcing the fovea's sensitivity to broader developmental disturbances. Together, these insights offer a framework for understanding the full spectrum of foveal morphology in health and disease and highlight the potential for genetic markers to inform diagnosis, prognosis, and future research in developmental macular disorders.

## Supplementary Material

Supplement 1

Supplement 2
