# Supplementary material for: Genome-Wide Insights Into the Genes and Pathways Shaping Human Foveal Development: Redefining the Genetic Landscape of Foveal Hypoplasia
Source: Invest Ophthalmol Vis Sci. 2025 Sep 9;66(12):22. doi: 10.1167/iovs.66.12.22 (PMC12425146; doi:10.1167/iovs.66.12.22)
Supplement: Supplement 1 [file iovs-66-12-22_s001.pdf]

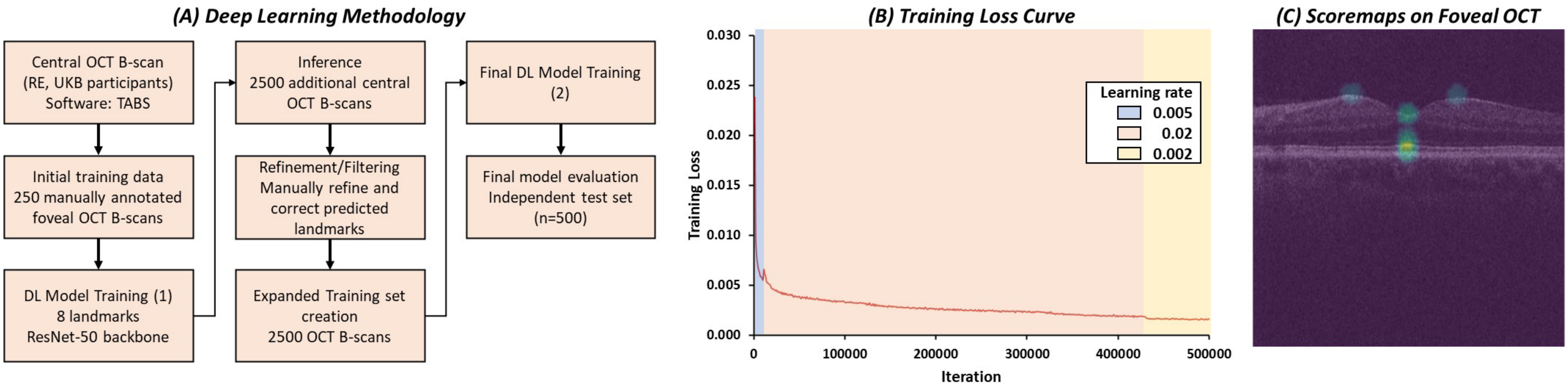

Supplementary figure 1: **Deep learning pipeline for automated landmark detection on foveal OCT scans.** (A) Overview of the model development pipeline. The central B-scan through the foveal centre was identified using the frame index from the Topcon Advanced Boundary Segmentation (TABS) software. An initial training set of 250 manually annotated B-scans was used to train a ResNet-50-based deep neural network (DeepLabCut v2.3.9) to detect eight anatomical landmarks: two ILM peaks, the foveal pit, the posterior inner retinal layer (IRL) boundary, and single points on the ELM, inner segment ellipsoid (ISe), RPE, and BM/choroid interface. The model was trained with data augmentation (including CLAHE, histogram equalisation, embossing, rotation, scaling). Predictions were made on 2,500 further scans, refined manually, and used to generate an expanded training set. Final model evaluation was performed on an independent test set (n = 500).(B) Training loss curve over 500,000 iterations. A staged learning rate schedule (0.005 → 0.02 → 0.002) was used. The model converged with final training loss ~0.0016.(C) Example foveal OCT scan with overlaid scoremaps. Warmer colours indicate higher prediction confidence. Shown here are landmark predictions used to calculate foveal pit depth, including ILM peaks and the foveal base. Deeper landmarks (e.g., ISe, RPE) were also reliably detected.

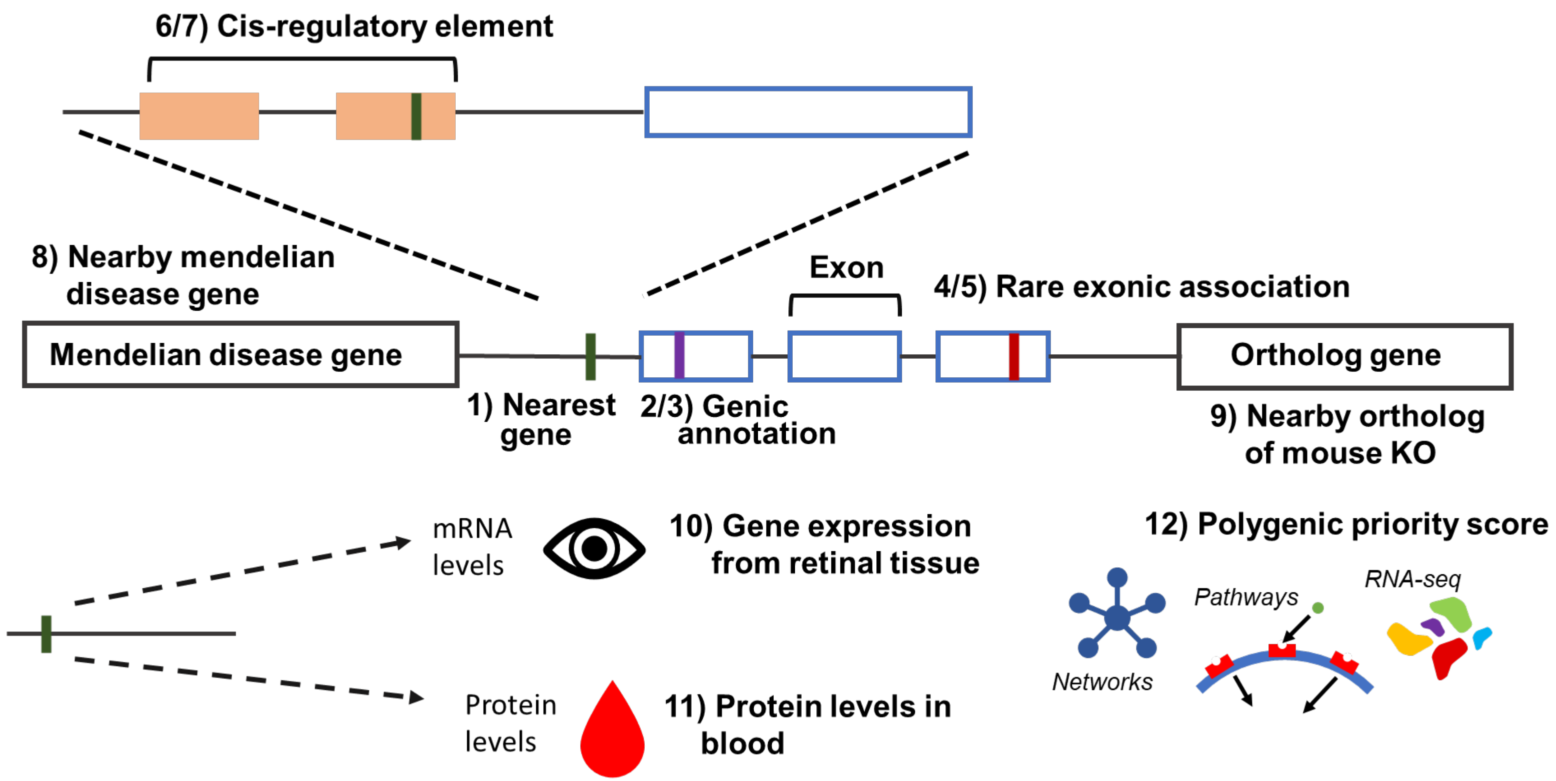

Supplementary Figure 2: **Schematic diagram illustrating the twelve lines of variant-to-gene evidence used to prioritise candidate genes from fine-mapped variants.**

For each fine-mapped variant, we systematically assessed nearby genes using the following twelve lines of evidence: **(1)** the nearest protein-coding gene (**within** ± 1 Mb) to the variant; **(2)** overlap of the variant with key gene features such as exons, promoters, or untranslated regions; **(3)** in silico pathogenicity predictions suggesting the variant may be deleterious; **(4)** presence of a significant single rare-variant association signal in a nearby gene (± 500Kb); **(5)** results from gene-based rare variant burden testing indicating a significant association (± 500Kb); **(6)** overlap of the variant with a cis-regulatory element (CRE) active in the developing neuroretina (± 250Kb); **(7)** overlap with a CRE active in the developing retinal pigment epithelium (± 250Kb); **(8)** proximity to a known Mendelian disease gene associated with a relevant foveal phenotype (± 500Kb); **(9)** proximity to a mouse orthologue with a relevant foveal phenotype in knockout models (± 500Kb); **(10)** overlap with a known retinal expression quantitative trait locus (eQTL) signal; **(11)** overlap with a known blood plasma protein quantitative trait locus (pQTL) signal in *cis*; and **(12)** genes with the highest polygenic priority scores (± 250Kb).
